# Double-balloon venous ethanol ablation for treatment of repetitive monomorphic ventricular complexes from intramural infero-basal septum: a case report

**DOI:** 10.1093/ehjcr/ytae216

**Published:** 2024-04-24

**Authors:** Andrea Rossi, Gianluca Mirizzi, Giancarlo Todiere, Alessia Gimelli, Martina Nesti

**Affiliations:** Tuscany Foundation ‘Gabriele Monasterio’, via Moruzzi, 1, 56100 Pisa, Italy; Tuscany Foundation ‘Gabriele Monasterio’, via Moruzzi, 1, 56100 Pisa, Italy; Tuscany Foundation ‘Gabriele Monasterio’, via Moruzzi, 1, 56100 Pisa, Italy; Tuscany Foundation ‘Gabriele Monasterio’, via Moruzzi, 1, 56100 Pisa, Italy; Tuscany Foundation ‘Gabriele Monasterio’, via Moruzzi, 1, 56100 Pisa, Italy

**Keywords:** Ventricular tachycardia, Premature ventricular complexes, Catheter ablation, Venous ethanol ablation, Alcoholization, Case Report

## Abstract

**Background:**

Ablation failures are common in case of intramural location of the arrhythmogenic substrate.

**Case summary:**

We report the case of a patient with cardiomyopathy contributed by frequent monomorphic ventricular arrhythmias (VAs) from intramural basal interventricular septum treated with double-balloon venous ethanol ablation (VEA) after a previous failed endocardial radiofrequency (RF) ablation.

**Discussion:**

Double-balloon VEA represents a safe and effective therapeutic option in case of intramural VAs also in the absence of venous collaterals joining selectively an intramural arrhythmic substrate.

**Learning points**

- Ablation failures are common in case of intramural ventricular arrhythmias due to inability to reach the arrhythmogenic substrate by conventional endocardial or epicardial ablation. Retrograde alcohol venous ablation using branches of coronary sinus was previously validated in humans and described in a variety of ventricular arrhythmias and aetiologies. The single balloon technique has several limitations, mostly due to the dimension and localization of the vein.
- The ‘double-balloon’ venous ethanol ablation technique (the first balloon used for ethanol injection and the second placed downstream to maintain ethanol between the two balloons) may overcome this limitation.

## Introduction

The ablation of premature ventricular complexes (PVCs) can represent a challenge for electrophysiologists, and different tools can be used to overcome these limits.

## Summary figure

**Figure:**
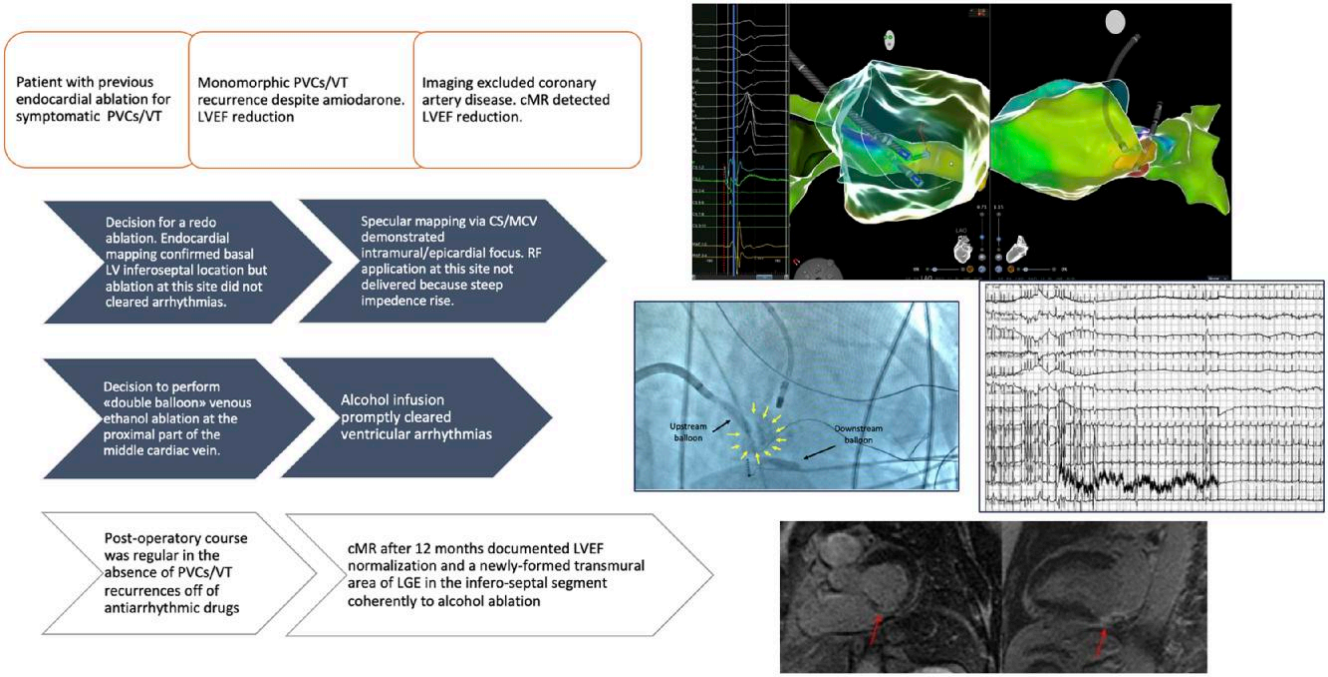


## Case presentation

A 63-year-old man with history of frequent monomorphic PVCs and non-sustained ventricular tachycardia (NSVT) despite previous endocardial ablation on the infero-basal septum, refractory to multiple antiarrhythmic drugs including amiodarone, presented for palpitations and dyspnoea. The physical examination was normal. No other pathological conditions and no family history of sudden death were referred.

**Figure 1:**
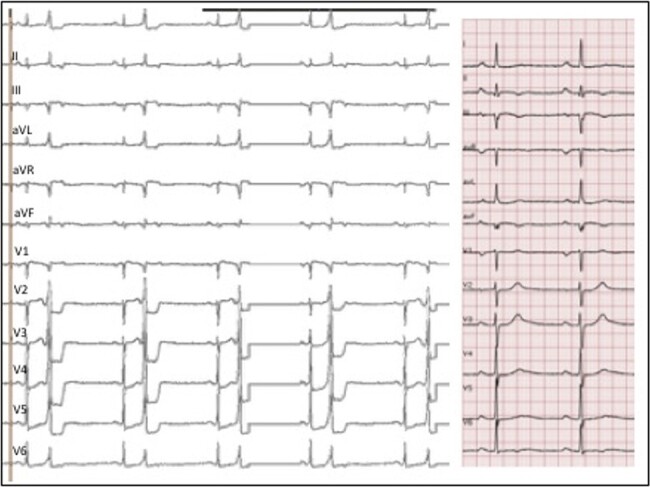
Twelve-lead electrocardiogram. On the left, 12-lead electrocardiogram at presentation. Right tracing shows a detail of normal sinus complexes.

The 12-lead electrocardiogram (ECG) pattern (*Figure 1*) showed a ventricular monomorphic bigeminy. Premature ventricular complexes had right bundle branch block morphology, R wave in lead I, an intermediate axis with II/III discordance. Normal sinus beats showed a first degree atrioventricular block (PR 210 ms), normal QRS and QTc interval, left QRS axis, and negative T-waves in infero-lateral leads. A 24 h Holter taken under d-sotalol 240 mg daily revealed 23 220 monomorphic PVCs (burden 23%) with ventricular couplets and runs of monomorphic NSVT (longest 25 beats). No CAD was detected by computed tomography. Cardiac magnetic resonance documented reduced global left ventricular ejection fraction (LVEF 45%) and increase of LV volume (113 mL/m^2^). Non-ischaemic areas of late gadolinium enhancement (LGE) were focally located in the basal infero-septal LV segment (site of previous endocardial catheter ablation) and in lateral LV wall (see Supplementary material).

**Figure 2:**
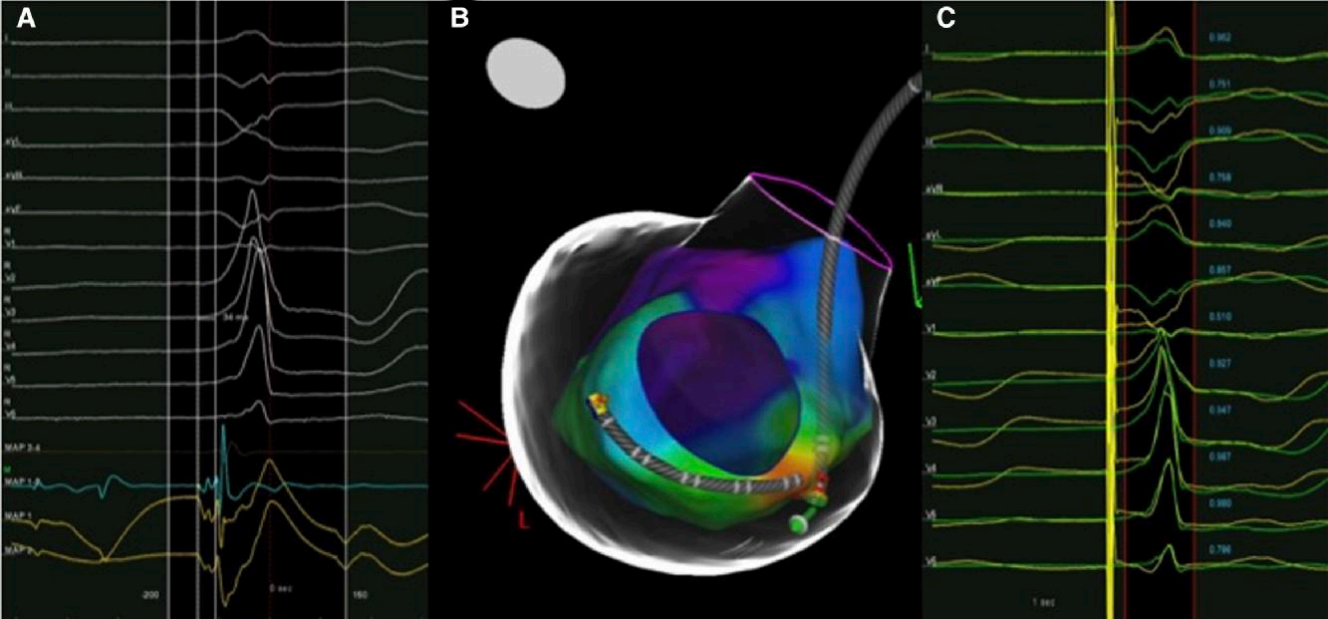
Left ventricular endocardial mapping. Local signal on the ablation catheter at the earliest endocardial location (*A*); activation map of the left ventricle shows the ablation catheter on the basal inferior septum and decapolar catheter in coronary sinus (*B*). Pacemap at the earliest activation site (*C*) demonstrates concordance between spontaneous PVC (green tracings) and paced complexes (yellow tracings).

**Figure 3:**
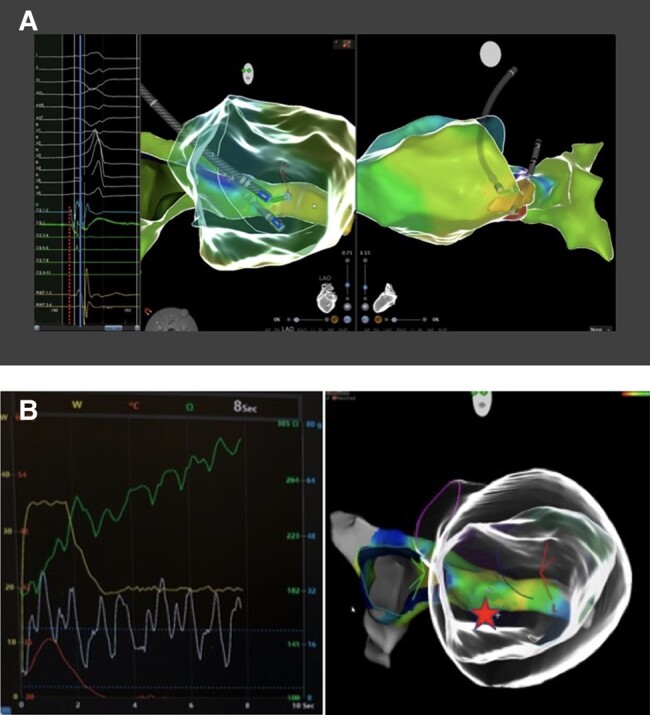
(*A*) Comparative endo-epicardial mapping. Electroanatomical map of the left ventricle and coronary venous system with ablation catheter at the endocardial earliest site and decapolar catheter at the epicardial earliest site (middle and right panels); 1–2 bipole on the decapolar catheter, at the emergence of middle cardiac vein, was earlier than ablation catheter signal (dashed red line) with unipolar QS configuration (left panel). (*B*) Steep impedance rise during radiofrequency ablation at the ostium of the middle cardiac vein. The right panel shows the anatomical map of coronary venous system and the anatomy of left ventricle (‘glass view’); the red star represents the site of radiofrequency ablation into the coronary venous system with a steep increase of impedance (left panel) and consequent energy delivery failure.

**Figure 4:**
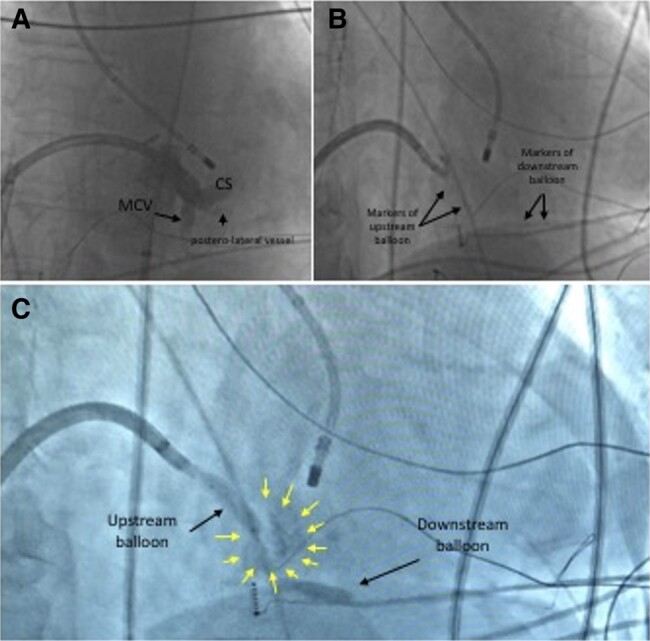
Venous ethanol ablation. Coronary sinus angiography through deflectable sheath (*A*) showed direction of middle cardiac vein (MCV). Balloons are positioned coherently with mapping and ablation catheter located at the left endocardial site as reference (*B*). Yellow arrows indicate extension of the contrast staining between balloons after alcohol delivery (*C*).

The patient was scheduled for a redo catheter ablation with CARTO3 system (Biosense Webster, Inc., Bar, CA, USA). LV activation map of PVCs confirmed the earliest endocardial activity (34 ms before the onset of QRS) at the infero-basal septum (*Figure 2A* and *B*) where the pacemap showed good concordance with the clinical PVC (*Figure 2C*). Radiofrequency (RF) ablation up to 45 W resulted in transient PVCs suppression. The specular site on the epicardial surface was mapped through the coronary venous system. The earliest activation (42 ms pre-QRS) with unipolar QS configuration was found at the proximal part of the middle cardiac vein (MCV) (*Figure 3A*). Due to a sudden impedance rise, we were not able to deliver RF at this site (*Figure 3B*). We decided to perform a venous ethanol ablation (VEA) using a ‘double-balloon’ technique. A selective coronary sinus (CS) angiography through an 8.5 Fr steerable sheath and subsequent venograms of the MCV through an 8 Fr left internal mammary artery catheter were obtained. Based on mapping data and venograms, a ‘downstream’ 3.5 × 12 mm angioplasty balloon was inflated up to 9 atm and an ‘upstream’ over-the-wire 4 × 20 mm balloon, inflated at 10 atm, was used to deliver ethanol. One millilitre of contrast medium was injected to evaluate the staining. We infused ethanol in 2 mL boluses each 3 min (total 10 mL) (*Figure 4*), monitoring local echogenicity with intracardiac echography. A rapid ventricular arrhythmia (VA) suppression was observed during alcohol delivery. Post-operatory course was regular, and continuous ECG monitoring showed no PVCs recurrence. The patient was discharged from hospital without antiarrhythmic drugs. After 12 months, no PVCs were observed at the 24 h Holter monitoring. LVEF improved up to 56%, and LV volume normalized (104 mL/m^2^). A newly formed transmural area of LGE was observed in the LV infero-septal segment, coherently to alcohol ablation site.

## Discussion

Catheter ablation is recommended in patients with cardiomyopathy suspected to be caused or contributed by frequent and predominantly monomorphic VAs.^1^ Ablation failures are common in case of intramural VAs due to inability to reach the arrhythmogenic substrate by conventional endocardial or epicardial ablation. Retrograde alcohol venous ablation using branches of CS was previously validated in humans and described in a variety of VAs and aetiologies.^2,3^ In an ideal scenario, an intramural vein joining the targeted substrate is cannulated and an over-the-wire balloon is used to block the downstream blood flux of the vein and to concentrate alcohol in the interested area. However, the single balloon technique has several limitations, mostly when the target vein is too large, the optimal signal recorded is proximal, if the ethanol is drained away from multiple venous branches or in the absence of intramural veins.^3^ The ‘double-balloon’ VEA technique (the first balloon used for ethanol injection and the second placed downstream to maintain ethanol between the two balloons)^4^ may overcome this limitation and can be used for extensive epicardial/intramural ablation.^3^ This case represents a detailed demonstration of acute effectiveness and durability of this technique as a bailout strategy for the treatment of VAs originating from an intramural septal substrate.

Double-balloon VEA represents a safe and effective therapeutic option in case of intramural VAs also in the absence of venous collaterals joining selectively an intramural arrhythmic substrate.

## Supplementary Material

ytae216_Supplementary_Data

## Data Availability

The data underlying this article are available in the article and in its online Supplementary material.

## Lead author biography

Andrea Rossi worked as a electrophysiologist at Fondazione Toscana Gabriele Monasterio in Pisa. His field of interest is ventricular arrhythmias and atrial fibrillation ablations.
