# Supplementary material for: Double-balloon venous ethanol ablation for treatment of repetitive monomorphic ventricular complexes from intramural infero-basal septum: a case report
Source: Eur Heart J Case Rep. 2024 Apr 24;8(5):ytae216. doi: 10.1093/ehjcr/ytae216 (PMC11095533; doi:10.1093/ehjcr/ytae216)
Supplement: ytae216_Supplementary_Data [file ytae216_supplementary_data.zip › data availability statement.docx]

The data underlying this article are available in the article and in its online supplementary material.
